# Gut Microbiota and Nutritional Profiles of Colon Cancer Patients Undergoing Chemotherapy: A Longitudinal Pilot Study

**DOI:** 10.3390/nu18030438

**Published:** 2026-01-29

**Authors:** Claire Han, Daniel Spakowicz, Diane Von Ah, Anne Noonan, Leah Pyter

**Affiliations:** 1 Center for Healthy Aging, Self-Management and Complex Care, College of Nursing, The Ohio State University, Newton Hall, 1585 Neil Avenue, Columbus, OH 43210, USA; vonah.1@osu.edu; 2 Cancer Control Program, The Ohio State University Comprehensive Cancer Center, Columbus, OH 43210, USA; 3 Division of Medical Oncology, Pelotonia Institute for Immuno-Oncology, The Ohio State University Comprehensive Cancer Center, Columbus, OH 43210, USA; daniel.spakowicz@osumc.edu; 4 GI Medical Oncology Section, The James: Cancer Treatment and Research Center, The Ohio State University, Columbus, OH 43210, USA; anne.noonan@osumc.edu; 5 Institute of Brain, Behavior and Immunology, The Ohio State University, Columbus, OH 43210, USA; leah.pyter@osumc.edu; 6 Department of Psychiatry and Behavioral Health, The Ohio State University, Columbus, OH 43210, USA

**Keywords:** colon cancer, fecal microbiota, diet, nutrition, chemotherapy

## Abstract

**Background/Objectives**: Nutrition and the gut microbiota influence treatment tolerance and recovery in patients with colon cancer receiving chemotherapy. This pilot study examined changes in diet quality and fecal microbiota over 6 months of chemotherapy and evaluated longitudinal associations between diet quality and gut microbiota diversity and taxa. **Methods**: 48 adults with stage II–III colon cancer receiving 5-fluorouracil-based chemotherapy were assessed at baseline and 6 months post-initiation. Diet quality was measured using 3-day dietary recalls to calculate Healthy Eating Index (HEI) scores. Stool samples underwent 16S rRNA sequencing to assess Shannon diversity, Beta and taxonomic composition. Pre–post changes were analyzed using paired tests, and associations between HEI and microbiota measures were evaluated using multivariable linear regression adjusting for demographic and clinical covariates. **Results**: Diet quality declined during chemotherapy, with reduced intake of fiber, fruits and vegetables, and whole grains. Gut microbial alpha diversity decreased over time. At the phylum level, *Actinobacteriota* decreased, while *Bacteroidota* and *Proteobacteria* increased. At the genus level, only *Streptococcus* (decreased) and *Escherichia* (increased) remained significantly altered after multiple testing correction. Higher baseline diet quality and improvements over time were associated with greater microbial diversity and lower *Proteobacteria* abundance. Diet quality was inversely associated with *Streptococcus* and *Escherichia* and positively associated with short-chain fatty acid-producing, fiber-responsive genera (*Faecalibacterium*, *Mediterraneibacter*, *Ruminococcus_E*, *Fusicatenibacter*). Baseline gut microbiota did not significantly associate with changes in diet quality. **Conclusions**: Chemotherapy was associated with declines in diet quality, gut microbial alpha diversity, along with shifts in beta diversity and microbial taxa. Higher diet quality appeared protective against microbial disruption, supporting a bidirectional relationship between diet and the gut microbiome during chemotherapy. Nutritional and microbiota-focused approaches warrant further investigation in the context of chemotherapy.

## 1. Introduction

Colon cancer is the third most commonly diagnosed cancer and the second leading cause of cancer-related deaths in the United States (US) [1], as well as globally [2]. In the United States alone, an estimated 106,590 new cases of colon cancer and 46,220 cases of rectal cancer will be diagnosed in 2025, with over 50,000 deaths expected annually due to colon cancer-related complications [3]. The incidence of colon cancer is particularly high among individuals over 50 years old, though rates are increasing among younger adults [4]. Standard treatment for stage II and III colon cancer typically involves surgical resection followed by adjuvant chemotherapy to prevent recurrence and improve survival [5].

Nutritional health plays a pivotal role in determining both treatment efficacy and health-related quality of life (HRQOL) in patients with colon cancer undergoing chemotherapy [6]. Malnutrition is prevalent among individuals receiving cancer treatments. Specifically, with estimates suggesting that up to 80% of gastrointestinal cancer patients experience nutritional deficiencies even before chemotherapy, as well as during or after chemotherapy [7]. Poor nutritional status can exacerbate treatment-related toxicity, reduce chemotherapy tolerance, increase hospitalization rates, impair physical function and HRQOL, and contribute to emotional distress and fatigue [8]. Furthermore, diet quality has been associated with better symptom control, fewer gastrointestinal side effects (e.g., diarrhea, mucositis, nausea), reduced infection risk, and improved immune and metabolic responses to therapy [9]. Good nutrition is thought to support mucosal repair and gut barrier integrity, which are often compromised during 5-fluouracil (FU)-based chemotherapy, which is the most common chemotherapy regimen in colon cancer [10]. Therefore, early identification of malnourished patients and timely nutritional intervention may improve clinical outcomes, reduce complications, and enhance adherence to the optimal chemotherapy regimens [11].

In recent years, gut microbiota have emerged as a critical mediator of chemotherapy response, toxicity, and patient-reported outcomes [12]. Chemotherapy for colon cancer causes significant changes in gut microbial composition and function, known as gut dysbiosis [13]. This dysbiosis has been associated with worse gastrointestinal and psychological symptoms, increased risk of adverse events, systemic inflammation, and reductions in function and HRQOL [14]. Numerous studies suggest that certain microbial taxa (e.g., Lactobacillus, Bifidobacterium) may protect against chemotherapy-induced toxicity, while others (e.g., Enterobacteriaceae, Clostridium difficile) may promote inflammatory or toxic effects in other cancer types [15]. Importantly, the relationship between the gut microbiota and nutrition is dynamic and could be bidirectional, forming a complex feedback loop that has significant implications for cancer treatment and recovery [16,17,18]. On one hand, diet is a key determinant of gut microbial diversity, composition, and function [18]. Diets rich in fiber, fruits, and whole grains are associated with increased microbial diversity and enhanced production of short-chain fatty acids (SCFAs), such as butyrate, which support gut epithelial integrity, modulate immune responses, and reduce inflammation [18]. Conversely, low-quality diets—often low in fiber and high in saturated fats and processed foods—can promote microbial imbalance and pro-inflammatory metabolites, leading to dysbiosis [19]. Mechanistically, the gut microbiota play an essential role in shaping nutritional status by influencing nutrient absorption, vitamin biosynthesis, bile acid metabolism, and even appetite regulation through gut-brain signaling pathways [20]. In the context of chemotherapy, microbial dysbiosis may exacerbate treatment-related toxicities by increasing intestinal permeability, impairing mucosal healing, and contributing to symptoms such as diarrhea, nausea, anorexia, and constipation [21,22]. These symptoms, in turn, can lead to reduced oral intake, malabsorption, and progressive nutritional decline, creating a vicious cycle of inflammation, gastrointestinal dysfunction, and undernutrition [23].

Despite the known clinical importance of both diet and microbiota in cancer health outcomes, including gastrointestinal conditions, chemotherapy side effects, function, and HRQOL, empirical data are lacking in patients with colon cancer [24]. Specifically, little is known about how diet quality and microbiota profiles change longitudinally in colon cancer patients during chemotherapy. Furthermore, fewer studies have examined their interdependence over time; that is, whether patients with worsening gut dysbiosis also experience declining dietary quality or vice versa [25,26]. This knowledge gap is particularly critical given the increasing recognition that nutrition-microbiota interactions may influence cancer outcomes, immune competence, HRQOL, and resilience to treatment [18,19]. However, few studies have explored how these factors change over time during chemotherapy in colon cancer patients. Understanding these longitudinal trajectories and whether specific populations are more vulnerable to adverse microbiota-nutrition dynamics, is critical for informing early, personalized interventions. Identifying patients with declining microbial diversity and worsening nutritional status during chemotherapy could guide timely nutritional support, such as high-fiber or protein-dense diets, or consideration of microbiota-modulating strategies (e.g., probiotics, prebiotics, or fecal microbiota transplantation). For example, patients with persistent constipation, poor appetite, taste alterations, or gastrointestinal discomfort may benefit from microbiota-targeted approaches to improve eating conditions and nutrient absorption.

Therefore, the objective of this study is twofold: (1) to characterize longitudinal changes in dietary quality and gut microbiota diversity and taxa during chemotherapy; and (2) to examine longitudinal associations between dietary quality and gut microbiota (baseline and changes over time from baseline to post 6 months chemotherapy initiation). Our primary hypotheses are: (a) Higher baseline diet quality will be positively associated with greater baseline gut microbial diversity and higher relative abundance of health-associated (favored) microbial taxa; and (b) declines in diet quality over time during treatment will be associated with reductions in gut microbial diversity and unfavorable shifts in health-associated microbial taxa over time. Our secondary hypotheses are: (a) Greater baseline gut microbial diversity and higher relative abundance of health-associated microbial taxa will be positively associated with higher baseline diet quality; and (b) lower baseline gut microbial diversity and reduced abundance of health-associated microbial taxa will be associated with greater declines in diet quality over time.

## 2. Materials and Methods

### 2.1. Design

We conducted a pilot study using a longitudinal, prospective study design in 48 patients with stages II or III colon cancer. All data were collected pre- and 6-month post chemotherapy initiation. Patients were enrolled at the Oncology Clinics of the Ohio State University Comprehensive Cancer Center between March 2024 and May 2025. This study was approved by the Ohio State University’s Institutional Review Boards (IRB), and all enrolled patients provided informed consent.

### 2.2. Sample

We used a purposive sampling method to recruit patients with stages II or III colon cancer (N = 48). Inclusion criteria for all participants included being ≥18 years old and having a histological proof of stages II-III colon cancer. In addition, patients had to be diagnosed with no distant metastasis, scheduled to receive 5-FU-based chemotherapy (5-FU alone or 5-FU combinations-FOLFOX or FOLFIRI), following colon surgeries, and had adequate organ function. Exclusion criteria for patients included chronic medical conditions involving the immune system (e.g., HIV, hepatitis B or C), regular use of immunosuppressive medications other than that routine steroids injection before the 5-FU-chemotherapy, those who cannot read and speak English, individuals with a current ostomy, chronic bowel disorders (such as irritable bowel syndrome); use of antibiotics, supplements of prebiotics, or probiotics within 1 month prior to each sample-collection time point; subjects with a significant cognitive or developmental delay; received neoadjuvant chemotherapy; and women who were pregnant.

### 2.3. Recruitment Strategies

Prior to participating in enrollment, the study protocol was approved by the Clinical Scientific Review Committee and the IRB of The Ohio State University Comprehensive Cancer Center. Patients with colon cancer were recruited through The Ohio State University Comprehensive Cancer Center–James Gastrointestinal Clinics and via mailings coordinated by the Ohio State University Cancer Center and College of Nursing, using purposive sampling methods. Recruitment was further supported through posters placed in the recruitment cancer center site. Patients who consented to being contacted for research received a phone call from a trained research assistant. Following completion of a screening questionnaire, eligible individuals were invited to review the study consent form, provide contact information, and schedule a follow-up phone call for enrollment and study orientation. To ensure diverse racial and ethnic representation, we purposively recruited racial and ethnic minority participants to comprise approximately half of the sample. This effort was supported through collaboration with The Ohio State University Center for Community Outreach and Engagement, which conducted targeted outreach to patients from underrepresented backgrounds.

### 2.4. Data Collection

Study materials and survey questionnaires were distributed either electronically via REDCap (Research Electronic Data Capture) or mailed directly to participants’ homes. Stool collection kits were also mailed, and instructions were reviewed with participants by phone. Participants were asked to return completed questionnaires either online or by mail using pre-stamped envelopes. Stool specimens were shipped overnight to the Ohio State University Biospecimen Services Shared Resource (OSU BSSR). Each participant provided diet quality data and stool samples at baseline before chemotherapy and again at 6 months, at the time of chemotherapy completion. Participants received a $25 electronic gift card for each completed time point, contingent upon receipt of both the questionnaire and stool sample. A total of 48 patients were retained from baseline to 6 months post-chemotherapy initiation, resulting in 100% retention with no dropouts across all data collection time points.

### 2.5. Measures

#### 2.5.1. Sociodemographic Data at Baseline

Demographic variables were collected via self-report survey or abstracted from electronic medical records, such as age, sex, marital status, employment status, race, education, types of insurance, and lifestyle.

#### 2.5.2. Clinical Data at Baseline

Clinical data were abstracted from electronic medical records, such as cancer stage, time since diagnosis, chemotherapy regimen, history of other cancer treatments, and comorbidities. The modified Charlson Comorbidity Index was used, which was derived from pre-treatment ICD-9/10 codes by summing weighted non-cancer comorbid conditions, excluding cancer diagnoses.

#### 2.5.3. Diet Quality (At Baseline and 6-Month Post-Chemotherapy Initiation)

We used the self-reported Short Form Food Frequency Questionnaire (SF-FFQ, 24 items, reliability = 0.74) and assessed this for 3 days at the time of data collection. We subsequently computed the mean values based on the 3-day dietary recalls [27]. Based on dietary intake and food groups including fruits, vegetables, grains, protein, dairy, fats and oils over the last week, we computed Healthy Eating Index (HEI) (0 “low” to 100 “high”) for use in our analysis, as a measure of diet quality (reliability = 0.91, sensitivity = 0.94) [28]. Nutritionist Pro software (version 2025) was used for data analysis [29].

#### 2.5.4. Fecal Microbiota Profile (At Baseline and 6-Month Post-Chemotherapy Initiation)

*At-Home Collection of Stool Samples.* Study participants were mailed at-home stool collection kits (DNAgenotek) with written and video instructions, return packaging, and return postage, for which 500 mg of stool was collected in 2 mL of DNA-stabilizer solution. Defecating normally, the participant used a sterile spatula to collect an aliquot and sealed the tube by screwing on the lid. Stool samples were collected using Zymo Research kits (Irvine, CA, USA) containing DNA/RNA Shield, which stabilizes microbial DNA at ambient temperature. Samples were shipped without freezing within 48 h of collection and processed upon receipt. Samples were then mailed directly to the OSU Biospecimen Services Shared Resource (OSU BSSR), and fecal samples were immediately stored at −80 °C. The OSU BSSR team sent the collected stool samples to Zymo Research (Irvine, CA, USA), where DNA extraction and sequencing were performed using 16S rRNA sequencing [30].

*Microbiota Profiling.* First, the DNA was purified with the Promega Maxwell RSC Fecal Microbiota kit (Promega, Madison, WI, USA), sequencing libraries were generated with the Illumina DNA Prep kit (Illumina, San Diego, CA, USA), the V3–V5 hypervariable regions of the bacterial 16S rRNA gene were amplified and 2 × 150 bp sequencing was performed on an Illumina NextSeq2000 at Zymo Research (Irvine, CA, USA) [30].

Then, we assessed diversity and taxa (abundance and types of bacterial taxa) of fecal microbiota bacterial profiles using 16S rRNA sequencing. Quality control was performed by Zymo Research and included positive microbial community controls, negative extraction and PCR controls, run-level monitoring with mock communities, and ASV-based bioinformatics processing with quality filtering and curated taxonomic databases [30]. Raw sequencing reads were quality filtered, trimmed, and denoised using the DADA2 plugin within QIIME 2, which performs error correction, chimera removal, and inference of ASVs. Taxonomic assignment was subsequently performed in QIIME 2 using a Sourmash approach with the Genome Taxonomy Data Base (GTDB v207) as the reference [30]. Relative abundances and alpha diversity metrics were calculated from normalized data. We excluded low-abundance bacteria with relative abundance levels < 2% at the level of phylum or genus for the statistical analyses. In this study, Shannon diversity (within-sample species diversity, calculated as a function of proportional abundance) was selected a priori as the primary alpha diversity measure. Based on the assumption of high concordance among commonly used alpha diversity metrics, as documented in prior ecological and microbiota literature, inclusion of additional indices (e.g., Simpson) was not expected to materially affect interpretation of the findings in this study [30]. Beta diversity was assessed using Bray–Curtis dissimilarity calculated from genus-level relative abundance data. Furthermore, our study focused on phylum and genus levels in microbiota abundance data by considering clinical or translational research purposes. We used QIIME 2 to compute Shannon diversity and bacterial taxonomic compositions (types and relative abundance).

### 2.6. Statistical Analysis

To evaluate within-subject changes in diet quality and stool microbiota alpha diversity between pre- and post-chemotherapy timepoints, paired statistical tests were conducted. Alpha diversity indices (e.g., Shannon) and diet scores (HEI) were assessed for normality using the Shapiro–Wilk test prior to selecting the appropriate test. Given that our data was non-normally distributed, we used Wilcoxon signed-rank tests and long-transformed prior to analysis. Within-subject dissimilarity was computed between paired pre- and post-chemotherapy samples. Differences in community composition by timepoint were evaluated using PERMANOVA with permutations constrained within subject. PCoA was used for visualization. Then, we used linear regression models to examine associations between the HEI and gut microbiota profiles. Both microbiota and HEI were assessed at baseline and as a change score (post-chemotherapy minus pre-chemotherapy). Microbiota outcomes included alpha diversity metrics and log-transformed relative abundances, evaluated at baseline and as change scores. All models were adjusted for age, sex, body mass index (BMI), cancer stage, and chemotherapy regimen (types and doses), and baseline HEI score or microbiota measures when applicable [24,25,26]. Results are reported as unstandardized B with 95% confidence intervals (CIs). Analyses were conducted in R (version 4.4.0) and Python (3.13) and significance was set at *p* < 0.05. For microbiota analyses involving multiple taxa, *p*-values were adjusted for multiple testing using the Benjamini–Hochberg false discovery rate (FDR) method, with *q* < 0.05 considered statistically significant.

## 3. Results

### 3.1. Characteristics of Participants

The study included 48 participants (mean age: 69.4 years, SD = 6.7; range: 59–86), with 54.2% male and 58.3% married (Table 1). Most were not employed (72.9%), and education levels varied: 31.3% had a high school diploma or less, 25.0% had some college, 29.2% had undergraduate degrees, and 14.5% had graduate degrees. The sample was evenly split between Black and White participants (50% each). Most had public insurance (58.3%), and 4.2% were uninsured. The majority were never smokers (62.5%), and 18.8% reported current alcohol use. About one-third reported low income (<$35,000), 37.5% middle, and 29.2% high income (≥$75,000). Healthy diet and routine physical activity were reported by 39.6% and 35.4%, respectively. Clinically, 76.9% had stage III and 23.1% stage II cancer. The mean time since diagnosis was 0.8 years (SD = 0.9). Chemotherapy included FOLFOX (50.0%), 5-FU alone (29.2%), and FOLFIRI (20.8%). All had prior colectomy, 25.0% received radiation. Most (95.4%) had a modified Charlson comorbidity index ≥ 2 (Table 1).

### 3.2. Nutritional Profiles Pre- and Post-Chemotherapy

Following chemotherapy, patients experienced marked reductions in several key dietary components (Table 2). Table 2 highlights substantial dietary deterioration during chemotherapy, particularly in plant-based nutrient sources and essential micronutrients. For macronutrients, the largest decline was observed in dietary fiber, which dropped by 17.1% (21.6 ± 5.4 to 17.9 ± 4.8 g/day, *p* = 0.009), followed by total calories (−12.7%, *p* = 0.012), total fat (−12.4%, *p* = 0.018), carbohydrates (−10.7%, *p* = 0.033), and protein (−10.5%, *p* = 0.021). For micronutrients, the greatest reduction occurred in vitamin D intake, which fell by 21.0% (310 ± 140 to 245 ± 115 IU, *p* = 0.041). Calcium and iron also showed notable decreases of 12.2% (*p* = 0.027) and 11.9% (*p* = 0.034), respectively. Changes in folate (−9.2%) and sodium (−0.7%) were not statistically significant. For dietary intake (food groups), the most pronounced change was in fruit and vegetable consumption, which declined by 26.5% (3.4 ± 1.2 to 2.5 ± 1.0 servings/day, *p* = 0.004). This was followed by whole grain intake (−23.1%, *p* = 0.007), added sugar (−16.2%, *p* = 0.026), and red meat (−18.2%, *p* = 0.048). The Healthy Eating Index (HEI) score significantly declined from 62.4 ± 8.5 pre-chemotherapy to 54.2 ± 9.3 post-chemotherapy (*p* = 0.015), reflecting a 13.1% reduction in overall dietary quality. This decrease was consistent with lower intake of fiber, fruits, vegetables, and whole grains, and reflects chemotherapy-related nutritional vulnerability.

### 3.3. Microbiota Profiles Pre- and Post-Chemotherapy

#### 3.3.1. Microbiota Alpha and Beta Diversity

**Figure 1:**
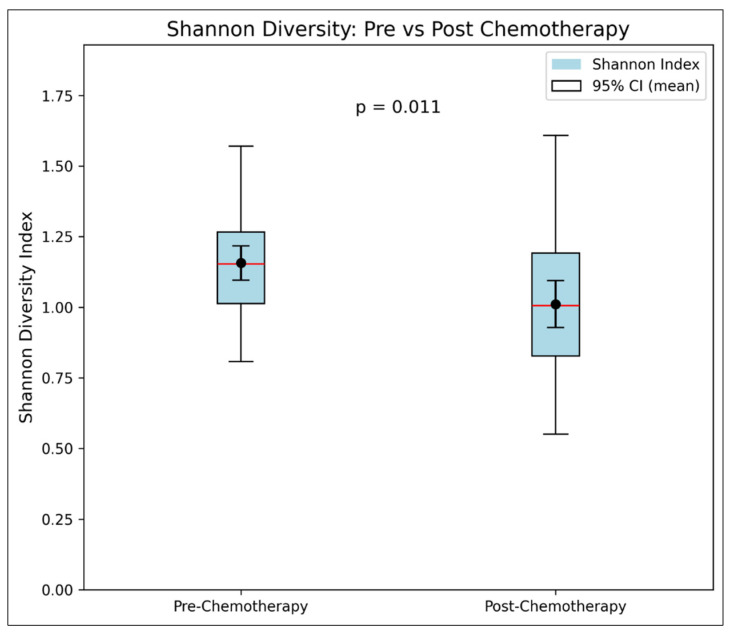
**Box Plot of Shannon Diversity (Pre–Post Chemotherapy).**

*Shannon Diversity (Alpha).* There is statistically significant evidence that chemotherapy reduces the Shannon diversity. The average value decreased significantly, from a pre-chemo mean of 1.20 (±0.20) to a post-chemo mean of 1.05 (±0.21) (*p* = 0.001, Table 3 and Figure 1).

**Figure 2:**
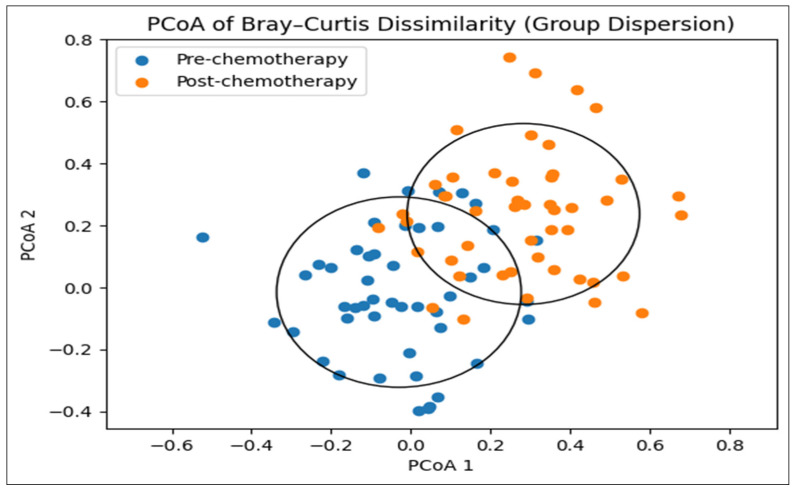
**Bray–Curtis beta diversity pre- and post-chemotherapy.**

*Beta Diversity*. Gut microbial community composition differed between pre- and post-chemotherapy samples. The median within-subject Bray–Curtis dissimilarity between paired samples was 0.34 (IQR 0.26–0.43), indicating modest compositional shifts over time. In paired PERMANOVA analyses with permutations constrained by participant, timepoint explained a small but significant proportion of variance in community composition (R^2^ = 0.03, *p* = 0.01). Consistent with this modest effect size, PCoA visualization showed partial overlap between pre- and post-chemotherapy samples, reflecting heterogeneous within-individual changes (Figure 2).

Principal coordinates analysis (PCoA) based on Bray–Curtis dissimilarity showing gut microbial community composition before and after chemotherapy. Circles represent group-level dispersion around the centroid for each timepoint, illustrating modest shifts in community composition over time.

#### 3.3.2. Relative Abundance (%) of Microbiota Phylum Pre- and Post-Chemotherapy

**Figure 3:**
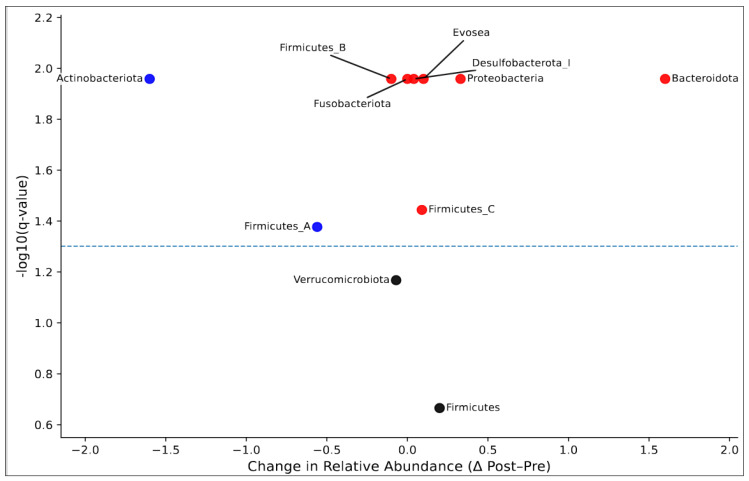
**Volcano plot of bacterial phylum-level changes pre- and post-chemotherapy.** Points represent pre- to post-chemotherapy changes (Δ Post–Pre) and −log10 *q*-values derived from paired Wilcoxon signed-rank tests with Benjamini–Hochberg false discovery rate (FDR) correction. Red and blue points indicate significantly increased and decreased phyla (*q* < 0.05), respectively, while black points denote non-significant changes. The dashed line indicates the nominal significance threshold (*p* = 0.05).

Eleven phyla, excluding extremely low baseline abundances (≤0.20%), were identified in our dataset. Based on Wilcoxon signed-rank tests comparing relative abundance before (Pre) and after (Post) an intervention, significant changes occurred in several bacterial phyla (Table 4 and Figure 3). *Actinobacteria* (Pre: 6.71%, Post: 5.11%, *q =* 0.011) showed a highly significant reduction over time. In contrast, *Bacteroidota* significantly increased (29.08% vs. 30.68%, *q =* 0.011), as did *Proteobacteria* (3.62% vs. 3.95%, *q =* 0.011) and *Firmicutes_C* (1.35% vs. 1.44%, *q =* 0.036). *Firmicutes_A* showed a marginal decrease (51.19% vs. 50.63%, *q =* 0.042).

#### 3.3.3. Relative Abundance (%) of Microbiota Genus Pre- and Post-Chemotherapy

**Figure 4:**
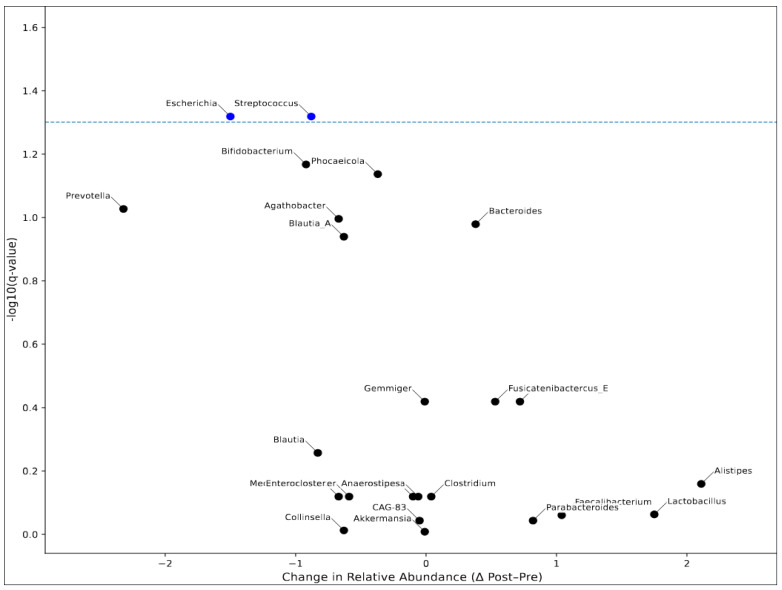
**Volcano plot of genus-level changes pre- and post-chemotherapy**. Points represent pre- to post-chemotherapy changes (Δ Post–Pre) and −log10 *q*-values derived from paired Wilcoxon signed-rank tests with Benjamini–Hochberg false discovery rate (FDR) correction. Blue points indicate significantly decreased genera (*q* < 0.05), respectively, while black points denote non-significant changes. The dashed line indicates the nominal significance threshold (*p* = 0.05).

Twenty-three genera were identified, excluding extremely low baseline abundances (≤0.20%). The Wilcoxon signed-rank test revealed significant changes in several bacterial genera from pre- to post-treatment based on *p*-values (Table 5 and Figure 4). At the genus level, only two taxa showed statistically significant changes after false discovery rate correction (*q* < 0.05) during the 6-month chemotherapy period. *Streptococcus* significantly decreased in relative abundance from pre- to post-chemotherapy (−22.2%, *q* = 0.048), whereas *Escherichia* significantly increased over time (+36.5%, *q* = 0.048). All other genera exhibited nominal pre–post changes but did not remain statistically significant after multiple testing correction.

### 3.4. Associations Between Diet Quality Measured with HEI and Microbiota Profiles

We further examined associations between diet quality (baseline and changes over time from baseline to 6 months post chemotherapy initiation) and alpha diversity and microbiota taxa (baseline and changes over time from baseline to 6 months post chemotherapy initiation), focusing on taxa with sufficient abundance (mean relative abundance ≥ 2%). Linear regression analyses revealed significant associations between diet quality (HEI scores) and both microbial alpha diversity and the relative abundances of specific phyla and genera (Table 6 and Table 7).

#### 3.4.1. Diet Quality and Shannon Diversity (Table 6)

Higher diet quality was significantly associated with greater gut microbial alpha diversity. Higher baseline HEI was positively associated with baseline Shannon diversity in adjusted models (B = 0.20, 95% CI 0.06–0.34, *p* = 0.02) and with increases in Shannon diversity over 6 months of chemotherapy (ΔM; B = 0.26, 95% CI 0.09–0.43, *p* = 0.01). Longitudinally, improvements in HEI were also associated with concurrent increases in Shannon diversity (B = 0.16, 95% CI 0.03–0.29, *p* = 0.02). In contrast, baseline Shannon diversity was not associated with subsequent pre–post changes in HEI (adjusted B = 0.75, 95% CI 0.03–1.03, *p* = 0.32).

#### 3.4.2. Diet Quality and Microbial Phyla (Table 6)

At the phylum level, higher baseline HEI was positively associated with baseline *Firmicutes* abundance (adjusted B = 1.35, 95% CI 0.15–2.55, *p* = 0.02), and this association remained significant after FDR correction (*q* = 0.049). Baseline HEI was also positively associated with increases in *Firmicutes* over time in adjusted models (B = 1.02, 95% CI 0.05–1.99, *p* = 0.03), although this association was borderline after FDR adjustment (*q* = 0.051). Associations between HEI and *Firmicutes* changes driven by ΔHEI were modest and did not remain significant after FDR correction (*q* = 0.075). *Firmicutes_A* showed positive associations with baseline HEI for baseline abundance (adjusted B = 0.98, 95% CI 0.02–1.94, *p* = 0.05) and for longitudinal change (B = 0.81, 95% CI 0.04–1.58, *p* = 0.04); however, these associations did not survive FDR correction (*q* = 0.067 and *q* = 0.053, respectively). No statistically significant associations were observed between HEI and *Bacteroidota* or *Actinobacteriota* at baseline or over time after FDR correction (all *q* > 0.10).

In contrast, diet quality demonstrated a robust inverse association with *Proteobacteria.* Higher baseline HEI was associated with lower baseline *Proteobacteria* abundance (adjusted B = −1.00, 95% CI −1.80 to −0.20, *p* = 0.01, *q* = 0.040) and with reductions in *Proteobacteria* over time (B = −0.87, 95% CI −1.68 to −0.06, *p* = 0.04, *q* = 0.045). Additionally, improvements in HEI were associated with concurrent decreases in *Proteobacteria* (B = −0.80, 95% CI −1.55 to −0.05, *p* = 0.04, *q* = 0.045).

#### 3.4.3. Diet Quality and Microbiota Genus (Table 7)

In genus-level bidirectional analyses of diet quality (HEI) and gut microbiota at baseline and over time, several consistent patterns emerged after covariate adjustment and FDR correction. Higher baseline HEI was associated with lower baseline abundance of *Blautia_A*, and longitudinal decreases in *Blautia_A* were linked to HEI improvement. Similarly, higher baseline HEI was associated with reductions over time in *Streptococcus*, *Escherichia*, *Prevotella*, *and Collinsella,* with corresponding decreases in these genera associated with improvements in HEI (all *q* ≤ 0.04).

SCFA-producing genera showed robust positive relationships with diet quality. Higher baseline HEI was associated with greater baseline abundance of *Faecalibacterium*, and increases over time were strongly linked to HEI improvement. Higher baseline HEI and increases in HEI were positively associated with increases in *Agathobacter*, *Alistipes, Mediterraneibacter*, *Ruminococcus_E*, and *Fusicatenibacter* (all *q* ≤ 0.03). No statistically significant associations were observed for *Bacteroides, Phocaeicola*, *Enterocloster*, or *Clostridium*.

## 4. Discussion

The current pilot study is the first to longitudinally examine diet quality and fecal microbiota profiles over time for 6 months chemotherapy in patients with colon cancer who underwent 5-FU-based chemotherapy regimens after colectomy. The primary objective was to describe and characterize alterations in fecal microbial diversity and taxonomic distribution at both the phylum and genus levels, and their potential correlations with baseline and changes in diet quality, as assessed by the HEI. We found that significant changes in dietary patterns (worsening diet quality) and relationships with the microbiota (lower Shannon diversity) occurred during chemotherapy. This suggested that chemotherapy played a role in decreasing adherence to the recommended dietary guidelines (e.g., a plant-forward diet emphasizing fruits, vegetables, whole grains, and legumes, while limiting red and processed meats, added sugars, and ultra-processed foods), and loss of microbial richness and evenness, which has been coupled with compromised gut health, with elevated risk of gastrointestinal and systemic complications [30,31,32,33]. Relationships between HEI scores and measures of the microbiota also suggest that the quality of the diet is deeply connected with the health of the gut microbiota. The baseline diet quality and the levels of change in HEI scores over time were correlated with variations in the microbial diversity and shifts in taxa, therefore implying that patients who maintained good diet plans may have been somewhat protected against microbiota disturbances [34,35]. Our findings shed light on chemotherapy’s impact on gut microbial ecology and eating behaviors, pointing to opportunities for supportive interventions, such as microbiota-focused therapies and nutrition strategies, to help reduce treatment-related adverse effects in colon cancer patients [36,37,38].

### 4.1. Diet Changes

Our findings show a clear decline in HEI scores after chemotherapy begins, indicating reduced diet quality among colon cancer patients. Declines were evident not only in the overall dietary quality score but also in key components, fruits, vegetables, and whole grains, with some participants shifting toward refined foods and lower fiber and protein intake. These patterns are in line with the nutritional issues reported among oncology patients in previous literature [39,40,41,42,43]; where diet quality commonly worsens during treatment due to physiological, psychological, and practical barriers during cancer treatments [41,42,43]. Gastrointestinal distress in patients with colon cancer is common and severe [41], including nausea, vomiting, mucositis, indigestion, diarrhea, and abdominal pain, limiting intake of nutrient-dense foods [41,42,43,44,45,46]. Fatigue and physical weakness further reduce the capacity to prepare balanced meals, leading many patients to rely on unhealthy processed foods [11,47]. Emotional distress may increase preference for energy-dense comfort foods [48,49], while financial strain related to cancer treatments can restrict access to fresh, high-quality options [50,51].

### 4.2. Microbiota Diversity

The Shannon index showed a significant reduction in alpha diversity after chemotherapy [52,53,54,55]. These findings are consistent with prior studies reporting chemotherapy-related dysbiosis and reduced alpha diversity in oncology populations [55,56,57,58,59,60], including breast cancer [58], hematopoietic stem cell transplant recipients [59] and pediatric cancer patients [60]. Mechanistically, cytotoxic therapy can damage the intestinal barrier, alter genomic integrity, and reduce ecological niches for commensal microbes [61,62,63]. Reduced diversity is linked to impaired barrier integrity, increased permeability, oxidative stress, DNA damage, and inflammation [61,62], contributing to infection risk, poor nutritional status, and delayed recovery. The small but significant PERMANOVA effect shown in Beta diversity, reflects modest chemotherapy-associated changes in gut microbial composition amid strong inter-individual variability. Evidence suggests that targeted interventions, including selective probiotics, optimized antibiotic strategies, dietary support, and other microbiota-directed therapies, may help restore diversity [64,65].

### 4.3. Microbiota Taxa

Chemotherapy was associated with significant shifts in gut microbiota composition at the phylum level, indicating broad ecological restructuring. *Actinobacteriota* decreased, a phylum that includes taxa involved in carbohydrate fermentation and SCFA production, potentially reflecting reduced dietary fiber intake and heightened sensitivity to chemotherapy-related gastrointestinal stress [66]. In contrast, *Bacteroidota* increased, consistent with their metabolic flexibility and ability to utilize host-derived substrates under conditions of altered nutrient availability. *Proteobacteria* also increased, a pattern commonly interpreted as a marker of microbial instability and epithelial stress, and may be driven by chemotherapy-induced inflammation, mucosal injury, or antibiotic exposure [66,67,68,69]. A modest decline in *Firmicutes_A*. Given the compositional nature of 16S rRNA sequencing data, these findings likely reflect relative redistribution of taxa rather than absolute changes in microbial abundance [66,67,68,69]. At the genus level, only two taxa showed significant changes after multiple testing correction. *Streptococcus* decreased during chemotherapy, which may reflect reduced oral–gut microbial transmission and lower availability of simple carbohydrates due to appetite loss [10]. In contrast, *Escherichia* increased, consistent with expansion of stress-tolerant, facultative anaerobes under inflammatory or oxygenated gut conditions [70,71]. In our study, the limited number of genus-level changes observed over the course of chemotherapy is biologically plausible. Chemotherapy represents a broad, non-specific perturbation to the gut ecosystem that preferentially alters higher-order microbial structure, while finer taxonomic shifts are more variable and subject to functional redundancy within genera. In addition, the compositional nature of 16S rRNA sequencing and modest sample size may further limit the detection of genus-specific change [72].

### 4.4. Associations Between Diet Quality (Measured as HEI) and Microbiota Profiles

Diet quality demonstrated a bidirectional relationship with the gut microbiota in this study, primarily through associations with overall microbial diversity and fiber-fermenting, SCFA-producing, and inflammation-associated taxa rather than widespread shifts across all microbial groups. Reduced intake of fiber-rich plant foods limits fermentation substrates for beneficial bacteria, contributing to lower SCFA-producing taxa and diminished microbial richness [73,74,75]. Consistent with this, higher baseline Healthy Eating Index (HEI) scores were associated with greater baseline Shannon diversity and smaller declines in diversity over time, suggesting that higher diet quality may confer microbial resilience during treatment [76,77,78,79]. At the phylum level, the most robust association after FDR correction was the inverse relationship between HEI and *Proteobacteria*. Expansion of *Proteobacteria* is commonly considered a marker of dysbiosis and inflammation; thus, lower *Proteobacteria* abundance among individuals with higher HEI may reflect improved gut homeostasis and reduced inflammatory stress during chemotherapy [78,79]. A reverse pattern was also observed in our study (Table 6): participants with higher dysbiosis tended to have worsening HEI scores during treatment, aligning with evidence that a well-balanced microbiota supports nutrient absorption, vitamin synthesis, and reduced gastrointestinal side effects [80]. At the genus level, diet quality was inversely associated with *Escherichia* and *Streptococcus*, taxa commonly linked to inflammatory or dysbiotic states, and positively associated with SCFA-producing, fiber-responsive genera, including *Faecalibacterium*, *Mediterraneibacter*, *Ruminococcus_E*, and *Fusicatenibacter*. Together, these findings support a diet–microbiota cross-talk model in which diet quality and microbial diversity mutually reinforce one another. Better baseline HEI fosters a more resilient microbial community as a protective factor, while greater microbial diversity helps sustain diet quality during treatment [81,82]. Clinically, these results underscore the importance of dietary counseling and microbiota-supportive strategies at the start of chemotherapy [83,84,85].

### 4.5. Clinical Implications

The bidirectional relationship between diet quality and the gut microbiota highlights the need to integrate nutritional and microbial assessments into supportive oncology care. Because diet quality, particularly fiber intake, declined over time in our cohort in other study in breast cancer [58], and this reduction was mirrored by losses in fiber-fermenting taxa (e.g., *Faecalibacterium*, *Mediterraneibacter*, *Ruminococcus_E*, and *Fusicatenibacters* in our study), assessing nutritional status and gut health before treatment may help identify patients at higher risk for microbiota disruption and treatment-related symptoms. Early nutritional rehabilitation, including counseling to increase fiber-rich foods and improve overall diet quality in compliance with the American Cancer Society nutritional guidelines, may strengthen microbial resilience before chemotherapy begins. Given that higher baseline HEI was associated with smaller declines in microbial diversity (Table 6), nutritional interventions should be initiated prior to chemotherapy and continued throughout treatment. For patients unable to meet fiber or diet-quality targets through self-management, safe adjunctive microbiota-supportive options for cancer patients with immunodeficiency, such as probiotics, prebiotics, symbiotics, or other evidence-based formulations, may help preserve beneficial taxa and mitigate dysbiosis associated with reduced fiber intake during chemotherapy. Maintaining diet quality, especially adequate fiber consumption, during chemotherapy is essential for reducing microbiota disruption, supporting gastrointestinal function, and improving symptom management. When feasible, fecal microbiota monitoring may help detect early microbial shifts linked to toxicity and guide timely intervention [58]. Overall, integrating nutritional assessment, individualized dietary counseling, and microbiota-directed strategies into routine cancer care may enhance functional resilience, reduce treatment side effects, and improve patients’ ability to tolerate and complete chemotherapy.

### 4.6. Strengths and Limitations

The study shows several strengths, including its design, assessment, analysis, and statistical models. The longitudinal design within the cancer and chemotherapy background allowed for observation of changes in both the diet quality and taxa of the gut microbiota throughout the whole process. Then, the integration of dietary assessments through the HEI scores with the profiling of microbiota levels provided a whole perspective of the patient’s gastrointestinal health. This analysis was not restricted but extended to both phylum and genus levels to identify specific taxa that may lead to protective effects or draw negative results.

However, some important limitations need to be acknowledged. Such constraints include the small size of the sample, which reduces the statistical power and the generalizability of study findings. Since the efforts were made to control for pre-specified covariates, some confounding ones, like the use of antibiotics that were not listed, concurrent medication and changes in them, and other co-morbidities, such as depression, cannot be ruled out. The analysis of the microbiota was limited to 16S rRNA sequencing, which excludes functional and metabolomics characterization, thereby restricting the findings and discussions. Furthermore, we acknowledge that reliance on a single alpha diversity metric is a limitation and may reduce comparability with studies reporting multiple diversity indices. Because all participants received chemotherapy, the independent effects of diet and chemotherapy on microbiota changes cannot be separated. Changes in gut microbiota are influenced by many factors beyond diet and chemotherapy, including antibiotic use, stress, body mass index, and other clinical characteristics. Therefore, the observed patterns likely reflect the combined influence of multiple co-occurring factors rather than a single exposure. Future larger studies with comparison groups and broader covariate assessment are needed to better clarify these relationships and account for additional social and clinical influences. Lastly, the dietary intake was self-reported, which might lead to the possibility of recall bias and misclassification.

## 5. Conclusions

Our pilot study shows that chemotherapy for colon cancer leads to parallel declines in diet quality and gut microbial health, marked by reduced HEI scores, lower microbial diversity, increased inflammatory genus (*Escherichia*) and losses of key fiber-fermenting and SCFA-producing taxa. Higher baseline diet quality and microbial diversity appeared protective, supporting a bidirectional diet–microbiota relationship throughout treatment. These findings suggest that early nutritional support and microbiota-focused strategies may help reduce treatment-related side effects. Larger studies are needed to validate these results and inform targeted dietary and microbiota-based interventions in oncology care.

## Figures and Tables

**Table 1:** **Sociodemographic and Clinical Characteristics of the Study Sample (N = 48).**

Characteristic	n (%) or Mean ± Standard Deviation (SD) [Range]
Demographic Characteristics	
Age (years)	69.4 ± 6.7 [59–86]
Sex	
Male	26 (54.2%)
Female	22 (45.8%)
Marital Status	
Married	28 (58.3%)
Single	20 (41.7%)
Body Mass Index	29.4 ± 3.2 [22.1–44.6]
Employment Status	
Employed	13 (27.1%)
Not Employed	35 (72.9%)
Education Level	
High School or Less	15 (31.3%)
Some College	12 (25.0%)
Undergraduate Degree	14 (29.2%)
Graduate Degree	7 (14.5%)
Race	
Black/White	24 (50.0%)/24(50.0%)
Insurance Type	
Private	18 (37.5%)
Public	28 (58.3%)
None	2 (4.2%)
Smoking Status	
Never Smoker	30 (62.5%)
Former Smoker	13 (27.1%)
Current Smoker	5 (10.4%)
Income Levels	
Low income (<$35,000/year)	16 (33.3%)
Middle income ($35,000–$74,999/year)	18 (37.5%)
High income (≥$75,000/year)	14 (29.2%)
Alcohol Use	
Yes/No	9 (18.8%)/39(81.2%)
Healthy Diet Adherence	
Yes/No	19 (39.6%)/29 (60.4%)
Routine Physical Activity	
Yes	17 (35.4%)
No	31 (64.6%)
Clinical Characteristics	
Colon Cancer Stage	
Stage II	11 (23.1%)
Stage III	37 (76.9%)
Years Since Diagnosis	0.8 ± 0.9 [0.3–4.0]
Chemotherapy Regimen	
FOLFOX (Folinic acid, Fluorouracil, and Oxaliplatin)	24 (50.0%)
5-FU (single agent)	14 (29.2%)
FOLFIRI (Folinic acid, Fluorouracil, and Irinotecan)	10 (20.8%)
History of Colectomy, Yes	48 (100.0%)
History of Radiation	
Yes	12 (25.0%)
No	36 (75.0%)
Modified Charlson Comorbidity Index (≥2)	46 (95.4%)

**Table 2:** **Nutritional Profiles Pre- and Post 6 months Chemotherapy (Paired comparison of 3-day averages of diet recall, N = 48).**

Component	Pre-Chemo (Mean ± SD)	Post-Chemo (Mean ± SD)	Change (Post–Pre)	% Change	*p*
**Macronutrients**					
**Total Calories (kcal)**	1925 ± 420	1680 ± 390	−245	−12.7%	**0.012**
**Protein (g)**	72.3 ± 15.8	64.7 ± 14.2	−7.6	−10.5%	**0.021**
**Carbohydrates (g)**	230.5 ± 50.3	205.8 ± 48.1	−24.7	−10.7%	**0.033**
**Total Fat (g)**	78.9 ± 16.7	69.1 ± 15.2	−9.8	−12.4%	**0.018**
**Fiber (g)**	21.6 ± 5.4	17.9 ± 4.8	−3.7	−17.1%	**0.009**
**Micronutrients**					
**Vitamin D (IU)**	310 ± 140	245 ± 115	−65	−21.0%	**0.041**
**Calcium (mg)**	925 ± 210	812 ± 185	−113	−12.2%	**0.027**
**Folate (mcg)**	380 ± 96	345 ± 84	−35	−9.2%	0.085
**Iron (mg)**	13.5 ± 3.2	11.9 ± 2.9	−1.6	−11.9%	**0.034**
**Sodium (mg)**	2300 ± 460	2285 ± 450	−15	−0.7%	0.770
**Water Intake (ml)**	1400 ± 310	1250 ± 285	−150	−10.7%	**0.045**
**Dietary Intake (Food Groups/Serving per day)**					
**Added Sugar (g)**	45.6 ± 14.9	38.2 ± 13.2	−7.4	−16.2%	**0.026**
**Red Meat (servings/day)**	1.1 ± 0.5	0.9 ± 0.4	−0.2	−18.2%	**0.048**
**Fruits and Vegetables (serving/day)**	3.4 ± 1.2	2.5 ± 1.0	−0.9	−26.5%	**0.004**
**Whole Grains (servings/day)**	2.6 ± 1.1	2.0 ± 0.9	−0.6	−23.1%	**0.007**
**HEI score (0–100), the higher the score,** **the better the diet quality**	62.4 ± 8.5	54.2 ± 9.3	−8.2	−13.1%	**0.015**

Note. A Wilcoxon signed-rank test was conducted to compare the nutritional profiles between pre- and post-treatment. A *p*-value of <0.05 is bolded and was considered statistically significant.

**Table 3:** **Alpha Diversity Index Pre- and Post 6 months Chemotherapy (Shannon Diversity, Paired comparison, N = 48).**

Metric	Pre-Chemotherapy	Post-Chemotherapy	*p*
**Mean ± SD**	1.20 ± 0.20	1.05 ± 0.21	0.011 for mean values
**Median [IQR]**	1.19 [1.05, 1.34]	1.03 [0.88, 1.20]	
**95% CI for mean**	[1.13, 1.27]	[0.97, 1.13]	

Note. A Wilcoxon signed-rank test was conducted to compare the Shannon diversity between pre- and post-treatment. A *p*-value of <0.05 was considered statistically significant.

**Table 4:** **Relative Abundance (%) of Microbiota Phylum Pre- and Post 6 months Chemotherapy (Paired comparison, N = 48).**

Phylum	Relative Abundance (%)			% Change	*p*	*q* (FDR)
	Pre%	Post%	Δ (Post − Pre)			
***Firmicutes_A***	51.19	50.63	−0.56	−1.1%	**0.019**	**0.042**
***Bacteroidota***	29.08	30.68	1.6	5.5%	**0.003**	**0.011**
***Firmicutes***	6.14	6.34	0.2	3.2%	0.131	0.216
***Actinobacteriota***	6.71	5.11	−1.6	−23.8%	**0.002**	**0.011**
***Proteobacteria***	3.62	3.95	0.33	9.1%	**0.003**	**0.011**
***Firmicutes_C***	1.35	1.44	0.09	6.6%	**0.013**	**0.036**
***Verrucomicrobiota***	1.19	1.12	−0.07	−5.8%	**0.037**	0.068
***Desulfobacterota_I***	0.20	0.24	0.04	20%	**0.002**	**0.011**
***Firmicutes_B***	0.02	0.02	0	0%	**0.002**	**0.011**
***Fusobacteriota***	0.01	0.01	0	0%	**0.002**	**0.011**
***Evosea***	0.01	0.01	0	0%	**0.002**	**0.011**

Note. A Wilcoxon signed-rank test was conducted to compare the total relative abundance of the top 14 most prevalent phyla between pre- and post-treatment. *p*-values were adjusted for multiple testing using the Benjamini–Hochberg false discovery rate (FDR) procedure. FDR-adjusted *q*-values < 0.05 were considered statistically significant. If the *p* and *q* values are < 0.05, they are bolded.

**Table 5:** **Relative Abundance (%) of Microbiota Genus Pre- and Post 6 months Chemotherapy (Paired comparison, N = 48).**

Genus	Relative Abundance (%)			% Change	*p*	*q* (FDR)
	Pre%	Post%	Δ (Post − Pre)			
***Bacteroides***	20.67	21.05	+0.38	+1.8%	**0.042**	0.105
***Blautia_A***	12.94	12.31	−0.63	−4.8%	**0.046**	0.115
***Phocaeicola***	9.62	9.26	−0.37	−3.8%	**0.021**	0.073
***Agathobacter***	6.06	5.40	−0.67	−11.0%	**0.039**	0.101
***Bifidobacterium***	5.55	4.64	−0.92	−16.5%	**0.019**	0.068
***Streptococcus***	3.95	3.07	−0.88	−22.2%	**0.012**	**0.048**
***Faecalibacterium***	3.46	4.50	+1.04	+30.0%	0.521	0.870
***Parabacteroides***	3.13	3.95	+0.82	+26.1%	0.663	0.905
***Alistipes***	2.19	4.30	+2.11	+96.5%	0.312	0.693
***Escherichia***	2.37	2.87	+0.50	36.5%	**0.012**	**0.048**
***Prevotella***	4.00	1.68	−2.32	−58.0%	**0.031**	0.094
***Enterocloster***	3.17	2.51	−0.67	−21.1%	0.412	0.760
***Collinsella***	2.78	2.15	−0.63	−22.6%	0.851	0.972
***Ruminococcus_E***	2.10	2.82	+0.72	+34.3%	0.122	0.381
***Fusicatenibacter***	1.93	2.46	+0.53	+27.6%	0.122	0.381
***Clostridium***	2.01	2.06	+0.04	+2.1%	0.412	0.760
***Gemmiger***	1.82	1.82	−0.01	−0.2%	0.122	0.381
***Roseburia***	1.71	1.65	−0.06	−3.5%	0.412	0.760
***Akkermansia***	1.64	1.62	−0.01	−0.9%	0.897	0.981
***Anaerostipes***	1.66	1.56	−0.10	−5.9%	0.412	0.760
***CAG-83***	1.50	1.45	−0.05	−3.5%	0.632	0.905
***Blautia***	1.82	0.99	−0.83	−45.7%	0.233	0.553
***Lactobacillus***	0.47	2.21	+1.75	+374.8%	0.512	0.865

Note. A Wilcoxon signed-rank test was conducted to compare the total relative abundance of the top 23 most prevalent genera between pre- and post-treatment. *p*-values were adjusted for multiple testing using the Benjamini–Hochberg false discovery rate (FDR) procedure. FDR-adjusted *q*-values < 0.05 were considered statistically significant. If the *p* and *q* values are <0.05, they are bolded.

**Table 6:** **Correlations Between Diet Quality (HEI) and Microbiota Profiles at Baseline and Changes from Baseline to Post 6 months Chemotherapy (Diversity and Microbiota Phylum).**

Diversity/Phyla	Input	Outcome	B (95% CI)	*p*	B (95% CI)	*p*	*q* (FDR)
**Shannon Diversity**	Baseline HEI	Baseline M	**0.23 (0.08, 0.38)**	**0.01**	**0.20 (0.06, 0.34)**	**0.02**	N/A
	Baseline HEI	Δ M	**0.31 (0.15, 0.48)**	**0.01**	**0.26 (0.09, 0.43)**	**0.01**	
	Baseline M	Δ HEI	0.78 (0.07, 1.15)	0.31	0.75 (0.03, 1.03)	0.32	
	ΔM	Δ HEI	**0.19 (0.07, 0.31)**	**0.01**	**0.16 (0.03, 0.29)**	**0.02**	
***Firmicutes_A***	Baseline HEI	Baseline M	**1.12 (0.03, 2.21)**	**0.04**	**0.98 (0.02, 1.94)**	**0.05**	**0.067**
	Baseline HEI	Δ M	0.67 (−0.40, 1.75)	0.21	**0.81 (0.04, 1.58)**	**0.04**	**0.053**
	Baseline M	Δ HEI	0.66 (−0.10, 1.42)	0.09	0.58 (−0.07, 1.23)	0.08	0.107
	ΔM	Δ HEI	0.61 (−0.18, 1.40)	0.13	0.55 (−0.15, 1.25)	0.12	0.160
***Bacteroidota***	Baseline HEI	Baseline M	0.88 (−0.05, 1.81)	0.07	0.75 (−0.12, 1.62)	0.09	0.120
	Baseline HEI	Δ M	0.55 (−0.47, 1.56)	0.29	0.60 (−0.25, 1.46)	0.15	0.200
	Baseline M	Δ HEI	0.52 (−0.20, 1.24)	0.15	0.45 (−0.20, 1.10)	0.17	0.227
	ΔM	Δ HEI	0.48 (−0.24, 1.20)	0.18	0.42 (−0.22, 1.06)	0.19	0.253
***Firmicutes***	Baseline HEI	Baseline M	**1.50 (0.30, 2.70)**	**0.02**	**1.35 (0.15, 2.55)**	**0.02**	**0.049**
	Baseline HEI	Δ M	0.91 (−0.27, 2.08)	0.13	**1.02 (0.05, 1.99)**	**0.03**	**0.051**
	Baseline M	Δ HEI	0.57 (−0.11, 1.25)	0.10	0.50 (−0.12, 1.12)	0.11	0.138
	ΔM	Δ HEI	**0.73 (0.01, 1.45)**	**0.05**	0.65 (0.01, 1.29)	0.06	0.075
***Actinobacteriota***	Baseline HEI	Baseline M	0.49 (−0.18, 1.16)	0.15	0.42 (−0.23, 1.07)	0.20	0.267
	Baseline HEI	Δ M	0.27 (−0.27, 0.81)	0.33	0.43 (−0.06, 0.91)	0.09	0.120
	Baseline M	Δ HEI	0.40 (−0.25, 1.06)	0.23	0.35 (−0.27, 0.97)	0.26	0.347
	ΔM	Δ HEI	0.31 (−0.32, 0.94)	0.33	0.25 (−0.34, 0.84)	0.40	0.533
***Proteobacteria***	Baseline HEI	Baseline M	**−1.11 (−1.86, −0.36)**	**0.01**	**−1.00 (−1.80, −0.20)**	**0.01**	**0.040**
	Baseline HEI	Δ M	**−0.94 (−1.67, −0.21)**	**0.03**	**−0.87 (−1.68, −0.06)**	**0.04**	**0.045**
	Baseline M	Δ HEI	**−0.95 (−1.70, −0.20)**	**0.02**	**−0.85 (−1.62, −0.08)**	**0.03**	**0.040**
	ΔM	Δ HEI	**−0.88 (−1.60, −0.15)**	**0.03**	**−0.80 (−1.55, −0.05)**	**0.04**	**0.045**

Note. Associations between Healthy Eating Index (HEI) and gut microbiota (M) diversity (Shannon diversity) and phylum-level taxa at baseline and over time (Δ = changes from baseline to 6 months post chemotherapy initiation) are presented as unadjusted and adjusted regression coefficients (Unstandardized B with 95% confidence intervals). Input variables indicate independent variables; outcome variables indicate outcomes in the regression models. Adjusted models controlled for baseline microbiota or baseline HEI, as appropriate, and covariates including age, sex, race, body mass index, cancer stage, and chemotherapy regimen. Shannon diversity analyses were pre-specified outcomes and were not adjusted for multiple testing. False discovery rate (FDR) correction using the Benjamini–Hochberg procedure was applied to phylum-level analyses to account for multiple comparisons, and FDR-adjusted *q*-values are reported for those associations.

**Table 7:** **Correlations Between Diet Quality (HEI) and Microbiota Profiles at Baseline and Changes from Baseline to Post 6 months Chemotherapy (Microbiota Genus).**

Genus	Input	Outcome	Unadjusted Models		Adjusted Models		
			B (95% CI)	*p*	B (95% CI)	*p*	*q* (FDR)
***Bacteroides***	Baseline HEI	Baseline M	+0.15 (−0.07, 0.37)	0.18	+0.10 (−0.12, 0.32)	0.37	0.28
	Baseline HEI	Δ M	+0.12 (−0.10, 0.34)	0.28	+0.08 (−0.14, 0.30)	0.47	0.37
	Baseline M	Δ HEI	+0.08 (−0.14, 0.30)	0.47	+0.05 (−0.17, 0.27)	0.65	0.47
	ΔM	Δ HEI	+0.21 (−0.03, 0.45)	0.09	+0.18 (−0.07, 0.43)	0.15	0.24
***Blautia_A***	Baseline HEI	Baseline M	−0.22 (−0.42, −0.02)	0.03	−0.18 (−0.38, 0.02)	**0.03**	**0.04**
	Baseline HEI	Δ M	−0.15 (−0.35, 0.05)	0.14	−0.12 (−0.32, 0.08)	0.24	0.19
	Baseline M	Δ HEI	−0.10 (−0.30, 0.10)	0.33	−0.07 (−0.27, 0.13)	0.49	0.33
	ΔM	Δ HEI	−0.25 (−0.47, −0.03)	0.03	−0.21 (−0.43, 0.01)	**0.03**	**0.04**
***Phocaeicola***	Baseline HEI	Baseline M	+0.10 (−0.12, 0.32)	0.37	+0.07 (−0.15, 0.29)	0.54	0.37
	Baseline HEI	Δ M	−0.05 (−0.27, 0.17)	0.65	−0.03 (−0.25, 0.19)	0.79	0.65
	Baseline M	Δ HEI	+0.03 (−0.19, 0.25)	0.79	+0.01 (−0.21, 0.23)	0.93	0.79
	ΔM	Δ HEI	−0.12 (−0.34, 0.10)	0.29	−0.09 (−0.31, 0.13)	0.42	0.37
***Agathobacter***	Baseline HEI	Baseline M	−0.18 (−0.38, 0.02)	0.08	−0.15 (−0.35, 0.05)	0.14	0.11
	Baseline HEI	Δ M	−0.22 (−0.42, −0.02)	0.03	−0.19 (−0.39, 0.01)	0.06	0.06
	Baseline M	Δ HEI	−0.07 (−0.27, 0.13)	0.49	−0.05 (−0.25, 0.15)	0.63	0.49
	ΔM	Δ HEI	−0.28 (−0.50, −0.06)	0.01	−0.24 (−0.46, −0.02)	**0.03**	**0.04**
***Bifidobacterium***	Baseline HEI	Baseline M	+0.19 (0.02, 0.36)	0.03	+0.15 (−0.02, 0.32)	0.09	0.09
	Baseline HEI	Δ M	+0.10 (−0.07, 0.27)	0.25	+0.07 (−0.10, 0.24)	0.42	0.25
	Baseline M	Δ HEI	+0.12 (−0.05, 0.29)	0.17	+0.09 (−0.08, 0.26)	0.30	0.23
	ΔM	Δ HEI	+0.16 (−0.01, 0.33)	0.06	+0.13 (−0.04, 0.30)	0.13	0.09
***Streptococcus***	Baseline HEI	Baseline M	−0.20 (−0.40, 0.00)	0.05	−0.16 (−0.36, 0.04)	0.12	0.07
	Baseline HEI	Δ M	−0.25 (−0.45, −0.05)	0.01	−0.21 (−0.41, −0.01)	**0.04**	**0.03**
	Baseline M	Δ HEI	−0.12 (−0.32, 0.08)	0.24	−0.09 (−0.29, 0.11)	0.38	0.24
	ΔM	Δ HEI	−0.30 (−0.52, −0.08)	0.004	−0.26 (−0.48, −0.04)	**0.02**	**0.02**
***Faecalibacterium***	Baseline HEI	Baseline M	+0.31 (0.08, 0.54)	0.01	+0.26 (0.03, 0.49)	**0.03**	**0.03**
	Baseline HEI	Δ M	+0.25 (0.02, 0.48)	0.04	+0.21 (−0.02, 0.44)	0.07	0.09
	Baseline M	Δ HEI	+0.18 (−0.05, 0.41)	0.12	+0.14 (−0.09, 0.37)	0.23	0.12
	ΔM	Δ HEI	+0.38 (0.14, 0.62)	0.002	+0.33 (0.09, 0.57)	**0.01**	**0.01**
***Parabacteroides***	Baseline HEI	Baseline M	+0.13 (−0.09, 0.35)	0.24	+0.09 (−0.13, 0.31)	0.41	0.24
	Baseline HEI	Δ M	+0.22 (0.00, 0.44)	0.05	+0.18 (−0.04, 0.40)	0.11	0.09
	Baseline M	Δ HEI	+0.15 (−0.07, 0.37)	0.18	+0.12 (−0.10, 0.34)	0.28	0.24
	ΔM	Δ HEI	+0.26 (0.02, 0.50)	0.03	+0.22 (−0.02, 0.46)	0.07	0.09
***Alistipes***	Baseline HEI	Baseline M	+0.11 (−0.11, 0.33)	0.32	+0.07 (−0.15, 0.29)	0.53	0.32
	Baseline HEI	Δ M	+0.35 (0.13, 0.57)	0.002	+0.31 (0.09, 0.53)	**0.006**	**0.01**
	Baseline M	Δ HEI	+0.14 (−0.08, 0.36)	0.21	+0.10 (−0.12, 0.32)	0.37	0.28
	ΔM	Δ HEI	+0.42 (0.18, 0.66)	0.001	+0.37 (0.13, 0.61)	**0.004**	**0.01**
***Escherichia***	Baseline HEI	Baseline M	−0.28 (−0.50, −0.06)	0.01	−0.24 (−0.46, −0.02)	**0.03**	**0.03**
	Baseline HEI	Δ M	−0.34 (−0.58, −0.10)	0.006	−0.29 (−0.53, −0.05)	**0.02**	**0.02**
	Baseline M	Δ HEI	−0.19 (−0.41, 0.03)	0.09	−0.15 (−0.37, 0.07)	0.18	0.09
	ΔM	Δ HEI	−0.41 (−0.65, −0.17)	0.001	−0.36 (−0.60, −0.12)	**0.003**	**0.01**
***Mediterraneibacter***	Baseline HEI	Baseline M	+0.17 (−0.05, 0.39)	0.12	+0.13 (−0.09, 0.35)	0.24	0.16
	Baseline HEI	Δ M	+0.29 (0.07, 0.51)	0.01	+0.25 (0.03, 0.47)	**0.03**	**0.03**
	Baseline M	Δ HEI	+0.14 (−0.08, 0.36)	0.21	+0.10 (−0.12, 0.32)	0.37	0.21
	ΔM	Δ HEI	+0.33 (0.09, 0.57)	0.004	+0.29 (0.05, 0.53)	**0.02**	**0.02**
***Prevotella***	Baseline HEI	Baseline M	−0.21 (−0.43, 0.01)	0.06	−0.18 (−0.40, 0.04)	0.11	0.08
	Baseline HEI	Δ M	−0.38 (−0.62, −0.14)	0.002	−0.34 (−0.58, −0.10)	**0.005**	**0.01**
	Baseline M	Δ HEI	−0.15 (−0.37, 0.07)	0.18	−0.12 (−0.34, 0.10)	0.29	0.18
	ΔM	Δ HEI	−0.44 (−0.68, −0.20)	0.001	−0.39 (−0.63, −0.15)	**0.002**	**0.01**
***Enterocloster***	Baseline HEI	Baseline M	−0.12 (−0.34, 0.10)	0.29	−0.09 (−0.31, 0.13)	0.42	0.24
	Baseline HEI	Δ M	−0.19 (−0.41, 0.03)	0.09	−0.15 (−0.37, 0.07)	0.18	0.12
	Baseline M	Δ HEI	−0.08 (−0.30, 0.14)	0.47	−0.05 (−0.27, 0.17)	0.65	0.47
	ΔM	Δ HEI	−0.23 (−0.47, 0.01)	0.06	−0.19 (−0.43, 0.05)	0.12	0.12
***Collinsella***	Baseline HEI	Baseline M	−0.26 (−0.48, −0.04)	0.02	−0.22 (−0.44, 0.00)	**0.05**	**0.04**
	Baseline HEI	Δ M	−0.31 (−0.55, −0.07)	0.01	−0.27 (−0.51, −0.03)	**0.03**	**0.03**
	Baseline M	Δ HEI	−0.14 (−0.36, 0.08)	0.21	−0.11 (−0.33, 0.11)	0.34	0.21
	ΔM	Δ HEI	−0.35 (−0.59, −0.11)	0.003	−0.30 (−0.54, −0.06)	**0.01**	**0.01**
***Ruminococcus_E***	Baseline HEI	Baseline M	+0.16 (−0.06, 0.38)	0.15	+0.12 (−0.10, 0.34)	0.29	0.20
	Baseline HEI	Δ M	+0.28 (0.06, 0.50)	0.01	+0.24 (0.02, 0.46)	**0.03**	**0.03**
	Baseline M	Δ HEI	+0.13 (−0.09, 0.35)	0.24	+0.09 (−0.13, 0.31)	0.41	0.24
	ΔM	Δ HEI	+0.32 (0.08, 0.56)	0.004	+0.28 (0.04, 0.52)	**0.02**	**0.02**
***Fusicatenibacter***	Baseline HEI	Baseline M	+0.18 (−0.04, 0.40)	0.11	+0.14 (−0.08, 0.36)	0.22	0.15
	Baseline HEI	Δ M	+0.31 (0.09, 0.53)	0.006	+0.27 (0.05, 0.49)	**0.02**	**0.02**
	Baseline M	Δ HEI	+0.15 (−0.07, 0.37)	0.18	+0.11 (−0.11, 0.33)	0.32	0.18
	ΔM	Δ HEI	+0.36 (0.12, 0.60)	0.003	+0.31 (0.07, 0.55)	**0.01**	**0.01**
***Clostridium***	Baseline HEI	Baseline M	−0.05 (−0.27, 0.17)	0.65	−0.03 (−0.25, 0.19)	0.79	0.65
	Baseline HEI	Δ M	+0.07 (−0.15, 0.29)	0.53	+0.05 (−0.17, 0.27)	0.65	0.65
	Baseline M	Δ HEI	+0.04 (−0.18, 0.26)	0.72	+0.02 (−0.20, 0.24)	0.86	0.72
	ΔM	Δ HEI	+0.09 (−0.13, 0.31)	0.42	+0.07 (−0.15, 0.29)	0.54	0.56

Note. Associations between baseline Healthy Eating Index (HEI) and gut microbiota (M) genus at baseline and over time (Δ = changes from baseline to 6 months post chemotherapy initiation), including unadjusted and adjusted regression coefficients (Unstandardized B with 95% CIs). Input variables indicate independent variables; outcome variables indicate outcomes in the regression models. Adjusted models account for baseline microbiota or baseline HEI when appropriate, and key covariates (i.e., age, sex, race, BMI, cancer stage, and chemotherapy regimen) when applicable. False discovery rate (FDR)-adjusted *q*-values were calculated using the Benjamini–Hochberg procedure within each genus. Δ indicates post–pre change. If the *p* and *q* values are <0.05, they are bolded.

## Data Availability

The data generated and analyzed during this study are not publicly available due to institutional policies and participant privacy protections, but de-identified aggregated data are available from the corresponding author upon reasonable request. Additional materials, including analytic code and study protocols, may also be shared upon request, contingent on appropriate data use agreements.

## Abbreviations

The following abbreviations are used in this manuscript:

5-FU	5-fluorouracil
16S rRNA	16S ribosomal ribonucleic acid
BMI	body mass index
CI	confidence interval
DNA	deoxyribonucleic acid
FOLFIRI	folinic acid, fluorouracil, and irinotecan
FOLFOX	folinic acid, fluorouracil, and oxaliplatin
GI	gastrointestinal
HEI	Healthy Eating Index
HIV	human immunodeficiency virus
HRQOL	health-related quality of life
IQR	interquartile range
IRB	Institutional Review Board
OSU BSSR	Ohio State University Biospecimen Services Shared Resource
QIIME 2	Quantitative Insights Into Microbial Ecology 2
REDCap	Research Electronic Data Capture
SCFA(s)	short-chain fatty acid(s)
SD	standard deviation
SF-FFQ	Short Form Food Frequency Questionnaire
US	United States
